# Structural Requirements for the Neuroprotective and Anti-Inflammatory Activities of the Flavanone Sterubin

**DOI:** 10.3390/antiox11112197

**Published:** 2022-11-07

**Authors:** Zhibin Liang, Pamela Maher

**Affiliations:** Cellular Neurobiology Laboratory, Salk Institute for Biological Studies, 10010 North Torrey Pines Road, San Diego, CA 92037, USA

**Keywords:** ferroptosis, oxytosis, inflammation, glutathione, Alzheimer’s disease

## Abstract

Alzheimer’s disease (AD) is the most frequent age-associated disease with no treatments that can prevent, delay, slow, or stop its progression. Thus, new approaches to drug development are needed. One promising approach is the use of phenotypic screening assays that can identify compounds that have therapeutic efficacy in target pathways relevant to aging and cognition, as well as AD pathology. Using this approach, we identified the flavanone sterubin, from Yerba santa (*Eriodictyon californicum*), as a potential drug candidate for the treatment of AD. Sterubin is highly protective against multiple initiators of cell death that activate distinct death pathways, potently induces the antioxidant transcription factor Nrf2, and has strong anti-inflammatory activity. Moreover, in a short-term model of AD, it was able to prevent decreases in short- and long-term memory. In order to better understand which key chemical functional groups are essential to the beneficial effects of sterubin, we compared the activity of sterubin to that of seven closely related flavonoids in our phenotypic screening assays. Surprisingly, only sterubin showed both potent neuroprotective activity against multiple insults as well as strong anti-inflammatory activity against several distinct inducers of inflammation. These effects correlated directly with the ability of sterubin to strongly induce Nrf2 in both nerve and microglial cells. Together, these results define the structural requirements underlying the neuroprotective and anti-inflammatory effects of sterubin and they provide the basis for future studies on new compounds based on sterubin.

## 1. Introduction

Alzheimer’s disease (AD) is the most frequent age-associated disease with no treatments that can prevent, delay, slow, or stop its progression. There are many reasons for the absence of an effective therapy, including the complexity of the disease and the paucity of efficient biomarkers for clinical trials. Since age is by far the major risk factor for AD and synapse loss and nerve cell death in AD are likely a consequence of many, cumulative toxic events, there is a strong rationale for a more clinically relevant approach to drug discovery based upon the biology of aging. Phenotypic screening assays that reflect multiple, age-associated neurotoxicity pathways rather than single molecular targets and which can identify compounds that have therapeutic efficacy in target pathways relevant to aging and cognition, as well as AD pathology are an effective approach. We have described a novel set of assays that represent distinct neurotoxicity pathways related to aging and neurodegenerative diseases [1,2,3]. In proof of principle studies, by using these assays in conjunction with plant-based starting materials, we identified a potential clinical candidate, the flavonoid fisetin [4,5]. However, given the complexity and heterogeneity of AD [6], it is likely that additional clinical candidates will be needed to effectively treat patients with the disease. Recently, we identified another potential drug candidate, the natural product sterubin, from Yerba santa (*Eriodictyon californicum*), a shrub native to California, USA, using our phenotypic screening approach [7]. Sterubin is highly protective against multiple inducers of cell death that activate distinct death pathways. Sterubin strongly induces the antioxidant transcription factor Nrf2 and also has robust anti-inflammatory activity [7]. Moreover, in a short-term model of AD using the amyloid beta (Aβ) peptide injected into the cerebral ventricles, it was able to prevent Aβ-induced decreases in short- and long-term memory [8]. Together, these biological activities suggest that sterubin and/or a derivative deserves further examination in the context of AD. As a prelude to these studies, we compared the activity of sterubin in our battery of innovative phenotypic screens relevant to the aging brain with a number of structurally closely related flavonoids in order to better understand which key chemical functional groups are essential to the beneficial effects of sterubin.

## 2. Materials and Methods

Materials: Sterubin was obtained from Michael Decker and the structure confirmed by NMR [8]. Eriodictyol (020056, lot#1902188), homoeriodictyol (021118S, lot#18092606), and luteolin (L-101, lot#0511112) were from Indofine (Hillsborough Township, NJ, USA). Sakuranetin (HYN3006, lot#122199) was from MedChemExpress (Monmouth Junction, NJ, USA), hydroxygenkwanin (S9205, lot#920501) was from Selleck Chemicals (Houston, TX, USA), hesperetin (10006084; lot#0461614-20) was from Cayman Chemicals (Ann Arbor, MI, USA), and chrysoeriol (1104S, lot#10) was from Extrasynthese (Genay, France). Erastin and RSL3 were from Cayman Chemicals. Bacterial lipopolysaccharide (LPS; L4524) and lipoteichoic acid (LTA; L2415) were from Sigma Aldrich (St. Louis, MO, USA). Interferon-gamma (IFNγ) was from Peprotech (Cranbury, NJ, USA). All other chemicals were from Sigma Aldrich or Thermo Fisher Scientific (Waltham, MA, USA).

Cell culture: HT22 mouse hippocampal nerve cells and MC65 human neuroblastoma cells were cultured in high glucose Dulbecco’s modified Eagle’s medium (DMEM) (Invitrogen, Thermo Fisher, Waltham, MA, USA) supplemented with 10% fetal calf serum (FCS) (Gibco, Thermo Fisher) and antibiotics and incubated at 37 °C in an atmosphere with 10% CO_2_. BV2 mouse microglial cells were grown in low glucose DMEM supplemented with 10% FCS and antibiotics and incubated under similar conditions.

### 2.1. Cell Culture Assays

Oxytosis/ferroptosis: 5 × 10^3^ HT22 cells were plated per well in 96 well plates. After 24 h of culture, the medium was exchanged with fresh medium and 5 mM glutamate, 500 nM erastin, or 250 nM RSL3 were added alone or in combination with the flavonoids at the indicated concentrations, as previously described [9,10]. Twenty-four hours later, the cellular viability was measured by the 3-(4,5-dimethylthiazolyl-2)-2,5-diphenyltetrazolium bromide (MTT) assay. In the absence of a protective compound ≥90% of the cells die under these conditions. In all cases, cells in the dishes were examined microscopically before the addition of the MTT reagent to ensure that any positive results in the MTT assay were not an artifact due to interaction of the flavonoids with the assay chemistry. In addition, equivalent results were obtained using the CyQuant Assay (Invitrogen, #C35011) following the manufacturer’s instructions.

Energy loss: HT22 cells were seeded onto 96 well plates as described under the oxytosis/ferroptosis assay. The medium was exchanged 24 h later with fresh medium and the cells were treated with 15 μM iodoacetic acid (IAA) alone (which results in 90–95% cell death) or in combination with the flavonoids at the indicated concentrations [9]. After 2 h, the medium was replaced with fresh medium without IAA but containing the flavonoids. Twenty-four hours later, the cellular viability was measured by the MTT assay.

Intracellular Aβ toxicity: MC65 cells [11] (from Bryce Sopher, University of Washington) express the C99 fragment of the amyloid precursor protein (APP) under the control of a tetracycline-sensitive promoter. The cells were routinely grown with 2 μg/mL tetracycline to block production of the C99 fragment [9]. For the assay, cells were plated at 1 × 10^5^ cells per well in 24 well tissue culture dishes and grown for 24 h. The next day, the cells were washed 3× with PBS and placed in Opti-minimal essential media (Opti-MEM, Invitrogen) in the presence (no induction) or absence (APP-C99 induced) of 2 μg/mL tetracycline in combination with the flavonoids. At day 3, the control cells in the absence of tetracycline were mostly dead and cell viability was determined by the MTT assay and confirmed by visual inspection.

H_2_O_2_ toxicity: HT22 cells were seeded onto 96 well plates as described under the oxytosis/ferroptosis assay. The medium was exchanged 24 h later with fresh medium and the cells were pre-treated with the flavonoids at the indicated concentrations for 60 min. H_2_O_2_ (1.5–2.5 mM) was then added to the wells and 24 h later, the cellular viability was measured by the MTT assay.

Inflammation: BV2 microglial cells were plated at 5 × 10^5^ cells/dish in 35 mm tissue culture dishes [12]. After culture overnight, the cells were treated with 25 ng/mL bacterial lipopolysaccharide (LPS), 10 µg/mL lipoteichoic acid, or 2.5 ng/mL interferon-gamma (IFNγ) alone or in the presence of the flavonoids. None of these treatments significantly affected cell viability after 24 h (viability: LPS = 98.6 ± 14%; LTA = 95.2 ± 15%; IFNγ = 104.0 ± 10%). After 24 h, the medium was removed, spun briefly to remove floating cells and 100 μL assayed for nitrite using 100 μL of the Griess Reagent in a 96 well plate. After incubation for 10 min at room temperature, the absorbance at 550 nm was read on a microplate reader. The absorbance was normalized to the cell viability as determined using the MTT assay. The levels of IL-6 and IL1β in the supernatants were determined using ELISAs (R&D Systems, Minneapolis, MN, USA) according to the manufacturer’s instructions.

### 2.2. Measurement of Total Glutathione (tGSH)

For measurement of tGSH, 3 × 10^5^ HT22 cells or 5 × 10^5^ BV2 cells were plated in 60 mm dishes. After 24 h of culture, the medium was exchanged with fresh medium and the indicated concentrations of glutamate (HT22 cells) or LPS (BV2 cells) and the flavonoids were added. The cells were treated for 24 h and then scraped into cold PBS and 10% sulfosalicylic acid was added at a final concentration of 3.3%. GSH was determined by the recycling assay based on the reduction of 5,5-dithiobis(2-nitrobenzoic acid) with glutathione reductase and NADPH [13] and normalized to protein recovered from the acid-precipitated pellet by treatment with 0.2 N NaOH at 37 °C overnight and measured by the bicinchoninic acid assay (Pierce, Rockford, IL, USA).

### 2.3. Western Blots

Sample Preparation: For Western blotting, 3 × 10^5^ HT22 cells or 5 × 10^5^ BV2 cells per 60 mm dish were grown for 24 h prior to the indicated treatments. For nuclear extracts, cells were rinsed twice in ice-cold Tris-buffered saline (TBS), scraped into ice-cold nuclear fractionation buffer (10 mM HEPES, pH 7.9, 10 mM KCl, 0.1 mM EDTA, 0.1 mM EGTA, 1 mM DTT, 1 mM Na_3_VO_4_, 1× protease inhibitor cocktail and 1× phosphatase inhibitor cocktail) and incubated on ice for 15 min. Then NP40 was added to a final concentration of 0.6%, the cells were vortexed and the nuclei pelleted by brief centrifugation. Nuclear proteins were extracted by sonication of the nuclear pellet in nuclear fractionation buffer and the extracts were cleared by additional centrifugation. Total protein extracts were prepared by rinsing the cells twice with ice-cold phosphate-buffered saline. The cells were scraped into lysis buffer (50 mM HEPES, pH 7.5, 50 mM NaCl, 50 mM NaF, 10 mM Na pyrophosphate, 5 mM EDTA, 1% Triton X-100, 1 mM Na_3_VO_4_, 1× protease inhibitor cocktail, 1x phosphatase inhibitor cocktail) and incubated on ice for 30 min. Extracts were sonicated and cleared by centrifugation. The supernatants were stored at −70 °C until analysis. Protein concentrations were quantified by the bicinchoninic acid method (Pierce) and adjusted to equal concentrations. Then, 5× Western blot sample buffer (74 mM Tris-HCl, pH 8.0, 6.25% SDS, 10% β-mercaptoethanol, 20% glycerol) was added to a final concentration of 2.5× and samples were boiled for 5 min.

Western blotting: For SDS-PAGE, equal amounts of cellular protein, typically 10–20 µg per lane, were used. All samples were separated using 4–12% Criterion XT Precast Bis-Tris Gels (Biorad, Hercules, CA, USA). Proteins were transferred to nitrocellulose membranes and the quality of protein measurement, electrophoresis and transfer checked by staining with Ponceau S. Membranes were blocked with 5% skim milk in TBS-T (20 mM Tris buffer pH 7.5, 0.5 M NaCl, 0.1% Tween 20) for 1 h at room temperature and incubated overnight at 4 °C in the primary antibody diluted in 5% BSA in TBS/0.05% Tween 20. The primary antibodies used were: rabbit anti-ATF4 (#sc-200, 1/500) and rabbit anti-Nrf2 (#sc-13032, 1/500) from Santa Cruz Biotechnologies (Dallas, TX, USA), rabbit anti-heme oxygenase 1 (#SPA-896, 1/10,000) from Stressgen (Victoria, BC, Canada), and guinea pig anti-p62 (#03-GP62-C, 1/10,000) from American Research Products (Belmont, MA, USA). Subsequently, blots were washed in TBS/0.05% Tween 20 and incubated for 1 h at room temperature in horseradish peroxidase-goat anti-rabbit (Biorad) or goat anti-guinea pig (Invitrogen) diluted 1/5000 in 5% skim milk in TBS/0.1% Tween 20. After additional washing, protein bands were detected by chemiluminescence using the Super Signal West Pico Substrate (Pierce). For all antibodies, the same membrane was re-probed for actin using HRP-conjugated rabbit anti-actin (#5125, 1/10,000) from Cell Signaling (Danvers, MA, USA). Autoradiographs were scanned using a Biorad GS800 scanner. Band density was measured using the manufacturer’s software. Relative protein expression was normalized to actin band density. Each Western blot was repeated at least three times with independent protein samples.

### 2.4. Transfection

For siRNA transfection, BV2 cells were plated in 60 mm dishes at 5 × 10^5^ cells/dish and 20 pmol Nrf2 siRNA (#sc-37049) from Santa Cruz Biotechnology or control siRNA (#1027280) from Qiagen (Hilden, Germany) were used along with RNAi Max (Invitrogen) according to the manufacturer’s instructions. The Nrf2 siRNA decreased Nrf2 expression by ~90% [7].

### 2.5. Calculation of Physicochemical Properties

The calculated physicochemical properties of the flavonoids were obtained using ChemBioDraw software and PubChem database https://pubchem.ncbi.nlm.nih.gov (accessed on 11 July 2022). The desired properties of central nervous system (CNS) druglikeness were evaluated on the basis of the previously published methods [14,15].

### 2.6. Statistical Analysis

Data from a minimum of three independent experiments were normalized, pooled, and analyzed using Graph Pad Prism 9 followed by the statistical tests indicated in the figure legends. *p* < 0.05 was taken as significant. The half maximal effective concentrations (EC_50_s) were determined from sigmoidal dose response curves also using GraphPad Prism 9.

## 3. Results

**Figure 1:**
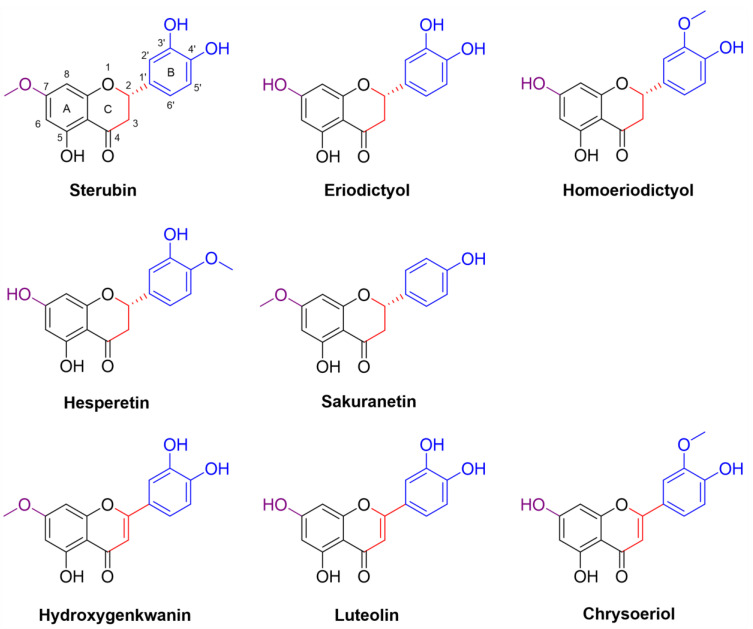
Structures of the eight flavonoids analyzed in this study. Colored structural features: 7-hydroxyl/methoxy on the A ring (purple), phenol on the B ring (blue), C2–C3 bond on the C ring (red).

For these studies, we compared eight structurally related flavonoids including sterubin, eriodictyol, homoeriodictyol, hesperetin, sakuranetin, hydroxygenkwanin (OHGK), luteolin, and chrysoeriol (Figure 1). All of these flavonoids except for sakuranetin have been found in *Eriodictyon* sp. extracts [16,17]. The major differences in the structures of these flavonoids are the presence or absence of a 7-methoxy group on the A ring, the absence or presence of a catechol group (with/without an *O*-methyl) on the B ring and whether or not the C ring is conjugated with a double bond between C2 and C3.

The compounds were first tested in the oxytosis/ferroptosis assay using two different system xc- inhibitors (glutamate and erastin) and RSL3, an inhibitor of glutathione peroxidase 4. As shown in Table 1, only sterubin and OHGK provided a high level of protection against all three of the insults with EC_50_s below 1 µM for both flavonoids. Both luteolin and eriodictyol were also protective against all three insults but with somewhat higher EC_50_s. Curiously, eriodictyol protected about as well as luteolin against glutamate and erastin but was much less effective than luteolin against RSL3. In contrast, none of the other flavonoids provided any protection against the three insults up to the highest concentration tested (10 µM). Similar results were obtained with protection against iodoacetic acid (IAA) which impairs energy metabolism and induces a form of cell death which has significant overlaps with oxytosis/ferroptosis [10].

As a complement to these assays, we also tested the compounds for protection against intracellular amyloid beta (Aβ) toxicity using the MC65 human nerve cell line. MC65 cells express the C99 fragment of the amyloid precursor protein (APP) under the control of a tetracycline-sensitive promoter [11]. When tetracycline is removed from the medium, the cells express the C99 fragment which is subsequently converted to Aβ by γ-secretase. The form of Aβ produced appears to be predominantly oligomeric Aβ_1–40_ [18,19]. The cells die within three days following Aβ aggregation within the cells. As shown in Table 1, and consistent with previous results [7], sterubin was highly effective against Aβ toxicity with an EC_50_ of 0.07 µM. Both luteolin and eriodictyol also were protective at sub-micromolar concentrations. Surprisingly, despite its robust effects against inducers of oxytosis/ferroptosis, OHGK was not very effective in the MC65 cells with an EC_50_ of 2.4 µM. All of the other flavonoids had EC_50_s ranging from 5 to >10 µM.

In addition, we tested the protective actions of the eight flavonoids against hydrogen peroxide (H_2_O_2_) which kills the cells by a pathway very distinct from oxytosis/ferroptosis [10]. As shown in Table 1, all of the flavonoids were much less effective against H_2_O_2_ as compared to the other insults tested. However, sterubin was still the most protective with an EC_50_ of 4.7 µM which is about 5-fold higher than its EC_50_ for protection against glutamate, erastin, RSL3 or IAA. Luteolin and eriodictyol were also ~5-fold less effective against H_2_O_2_. In contrast, OHGK was ~25-fold less effective against H_2_O_2_. Similar results (not shown) were obtained if the flavonoid-containing medium was replaced with fresh medium with no flavonoid prior to the addition of H_2_O_2_ indicating that the flavonoids that protect against H_2_O_2_ are not directly interacting with the oxidant in the medium (not shown). 

**Figure 2:**
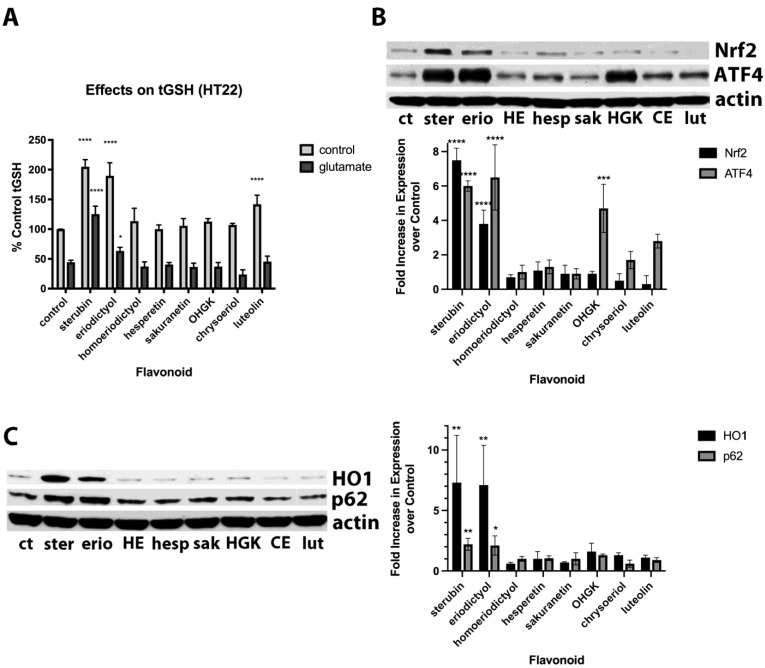
(**A**) Comparison of the effects of 10 µM sterubin, eriodictyol, homoeriodictyol, hesperetin, sakuranetin, OHGK, chrysoeriol, or luteolin on basal tGSH levels and tGSH levels in the presence of 5 mM glutamate. tGSH levels were measured after 24 h with a chemical assay. Results are the average of three independent experiments. **** *p* < 0.0001 relative to control for cells not treated with glutamate; **** *p* < 0.0001, * *p* < 0.01 relative to cells treated with glutamate alone. One-way ANOVA followed by Tukey’s post test analysis. (**B**) HT22 cells were treated with 10 µM sterubin, eriodictyol, homoeriodictyol, hesperetin, sakuranetin, OHGK, chrysoeriol or luteolin for 4 h. Nuclei were prepared and examined by Western blotting for Nrf2 and ATF4. Representative blots are shown. Quantification of the results from 3 independent experiments is shown. The expression of each protein was normalized to the level of actin in the sample and the results are presented relative to the controls which were set at 1. **** *p* < 0.0001, *** *p* < 0.001 relative to control. One-way ANOVA followed by Tukey’s post test analysis. (**C**) HT22 cells were treated with 10 µM sterubin, eriodictyol, homoeriodictyol, hesperetin, sakuranetin, OHGK, chrysoeriol or luteolin for 8 h and whole cell extracts examined for two markers of Nrf2 activation, heme oxygenase 1 (HO-1) and p62, by Western blotting. Representative blots are shown. Quantification of the results from 3 independent experiments is shown. The expression of each protein was normalized to the level of actin in the sample and the results presented relative to the controls which were set at 1. ** *p* < 0.01, * *p* < 0.05 relative to control. One-way ANOVA followed by Tukey’s post test analysis.

Previously, we showed that sterubin both greatly increased basal total GSH (tGSH) levels and also maintained tGSH levels in the presence of glutamate and this contributed to its beneficial effects [7], so we next looked at the impacts of the different flavonoids on tGSH levels in the absence and presence of glutamate. To allow us to assay tGSH in glutamate-treated cells, we used glutamate concentrations that lowered tGSH levels by ~50% and therefore did not result in significant cell death. As shown in Figure 2A, sterubin, eriodictyol and, somewhat less effectively, luteolin, all increased basal tGSH levels. Both sterubin and, to a much lesser extent, eriodictyol also maintained tGSH levels in the presence of glutamate, consistent with our previous report [7]. However, none of the other flavonoids, including both luteolin and the highly protective OHGK, were able to maintain tGSH levels in the presence of glutamate.

Given that we previously showed that sterubin could induce Nrf2 and that this induction contributed to its ability to increase tGSH levels and protect against glutamate [7], we next looked at the effects of the eight flavonoids on Nrf2 induction. As shown in Figure 2B, while sterubin and eriodictyol induced nuclear Nrf2, none of the other flavonoids did so. However, sterubin, eriodictyol, luteolin, and OHGK all induced nuclear ATF4 which also plays a role in the response of cells to oxidative stress [20]. To support these results regarding Nrf2 induction, we also examined the effects of the compounds on the production of several Nrf2-regulated proteins including HO1 and p62. As shown in Figure 2C, and consistent with the effects of the compounds on nuclear Nrf2 induction, only sterubin and eriodictyol increased the levels of these two proteins.

Since sterubin has potent anti-inflammatory activity in microglial cells [7] and this could be an important aspect of its overall protective effects in vivo, we also compared its anti-inflammatory activity to that of the other flavonoids. For these experiments, we used BV2 microglial cells treated with three different inducers of an inflammatory response including the Toll like receptor 4 (TLR4) agonist bacterial lipopolysaccharide (LPS), the TLR1/2 agonist lipoteichoic acid (LTA) and interferon-gamma (IFNγ). We tested different inducers in order to determine if there were distinct structural requirements for protection against pro-inflammatory stimuli that act on different targets. As readouts, we looked at both nitric oxide (NO) and cytokine (IL6 and IL1β) production (Table 2). Sterubin was not only highly effective against LPS-induced production of both NO and cytokines, as we showed previously [7], but it was also equally effective against LTA-induced NO and cytokine production. However, while it also reduced IFNγ-induced NO and IL6 production (IFNγ did not increase IL1β levels), it was 4–5 fold less effective than against LPS and LTA. Both eriodictyol and luteolin were also effective against LPS and LTA-induced NO and cytokine production at the concentrations tested (100 nM–10 µM) but had little or no effect on the pro-inflammatory response to IFNγ. The other five flavonoids either had no effect or only a small effect on LPS-, LTA- or IFNγ-induced NO or cytokine production at these same concentrations. Most striking was the very low anti-inflammatory activity shown by OHGK against all three pro-inflammatory stimuli despite its strong protective effects against oxytosis/ferroptosis.

**Figure 3:**
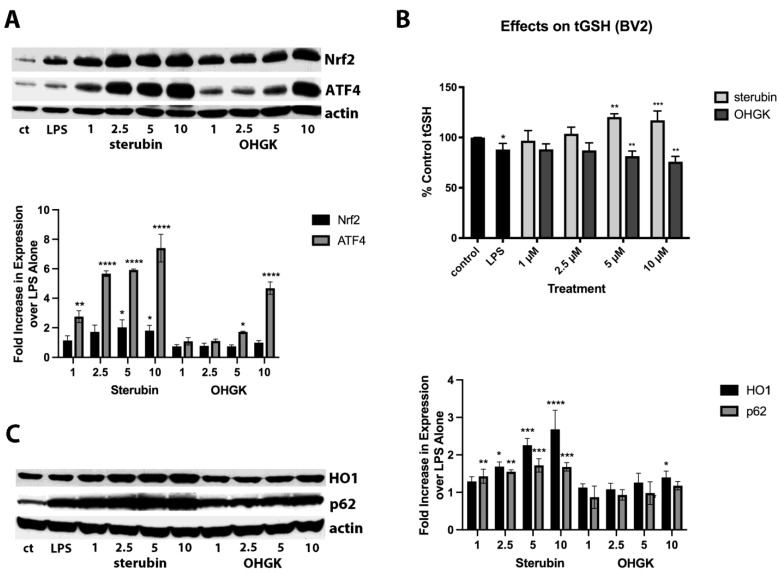
(**A**) BV2 cells were treated with 1–10 µM sterubin or OHGK for 4 h in the presence of 25 ng/mL LPS. Nuclei were prepared and examined by Western blotting for Nrf2 and ATF4. Representative blots are shown. Quantification of the results from 3 independent experiments is shown. The expression of each protein was normalized to the level of actin in the sample and the results are presented relative to LPS which was set at 1. **** *p* < 0.0001, ** *p* < 0.01, * *p* < 0.05 relative to LPS treatment alone. One-way ANOVA followed by Tukey’s post test analysis. (**B**) Comparison of the effects of 1–10 µM sterubin or OHGK on tGSH levels in BV2 cells treated with LPS (25 ng/mL). tGSH levels were measured after 24 h with a chemical assay. Results are the average of three independent experiments. *** *p* < 0.001, ** *p* < 0.01, * *p* < 0.05 relative to control. One-way ANOVA followed by Tukey’s post test analysis. (**C**) HT22 cells were treated with 1–10 µM sterubin or OHGK for 8 h in the presence of LPS (25 ng/mL) and whole cell extracts examined for two markers of Nrf2 activation, heme oxygenase 1 (HO-1) and p62, by Western blotting. Representative blots are shown. Quantification of the results from three independent experiments is shown. The expression of each protein was normalized to the level of actin in the sample and the results are presented relative to LPS which was set at 1. **** *p* < 0.0001, *** *p* < 0.001, ** *p* < 0.01, * *p* < 0.05 relative to LPS alone. One-way ANOVA followed by Tukey’s post test analysis.

Because of the high structural similarity to sterubin but with the presence of a conjugated double bond between C2 and C3, we decided to focus on the difference between the protective and anti-inflammatory effects of OHGK in further studies. Since we previously showed that the ability of sterubin to induce the transcription factor Nrf2 played a key role in its anti-inflammatory effects [7] and, in the HT22 cells, we saw no induction of Nrf2 by OHGK, we reasoned that this could explain its lack of anti-inflammatory activity. Indeed, as shown in Figure 3A, OHGK was unable to induce nuclear Nrf2 accumulation at any of the doses tested (1–10 µM) in the BV2 cells although it did induce ATF4. This absence of Nrf2 induction correlated with a lack of an effect of OHGK on the levels of tGSH as well as the Nrf2 target proteins HO1 and p62 (Figure 3B,C) which contrasted with the robust effects of sterubin on all of these parameters.

**Figure 4:**
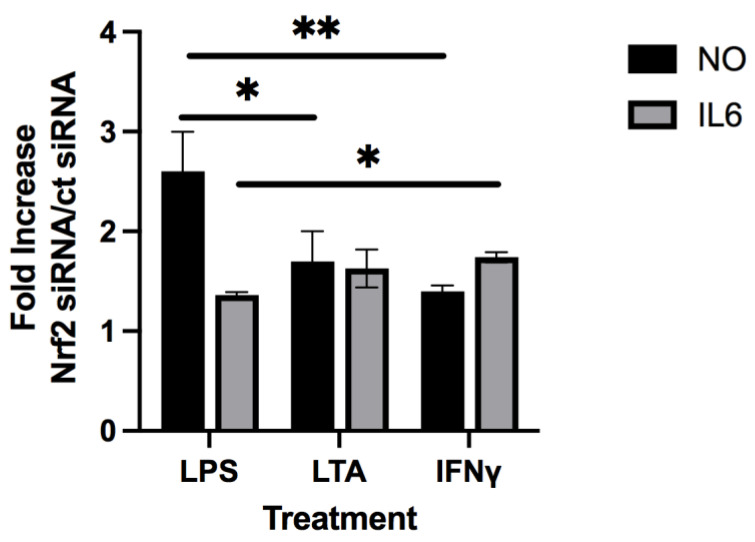
BV-2 cells were transfected with control siRNA or Nrf2 siRNA 24 h before seeding. Transfected BV2 cells were treated overnight with 25 ng/mL LPS, 10 µg/ml LTA or 25 ng/ml IFNγ alone or in the presence of 10 µM sterubin. Cell culture supernatants were cleared and assayed for NO by the Griess assay or IL6 using an ELISA. Results are presented as the ratio of the percent inhibition of NO or IL6 production by sterubin in the presence of Nrf2 siRNA to the percent inhibition in the presence of control siRNA. Thus, a decrease in the inhibition of NO or IL6 production in the presence of Nrf2 siRNA leads to a higher fold increase in the ratio. The results are the average of three independent experiments. * *p* < 0.05, ** *p* < 0.01 using the *t*-test.

Since OHGK appeared to have some anti-inflammatory activity against IFNγ with respect to inhibition of IL6 production while sterubin was significantly less effective against IFNγ as compared to LPS and LTA, we decided to look further at the role of Nrf2 in the anti-inflammatory effects of sterubin against LTA and IFNγ. For these experiments, BV2 cells were treated with the control or Nrf2 siRNA and then the ability of sterubin to reduce the production of both NO and IL6 in response to the three pro-inflammatory agents was examined. As shown in Figure 4, while Nrf2 knockdown strongly reduced the ability of sterubin to prevent NO induction by LPS and thereby increased NO production 2.6 fold over that seen with control siRNA as we reported previously [7], it had a significantly lesser effect on sterubin’s ability to dampen NO production in response to both LTA and IFNγ. Curiously, and in contrast to our initial assumption, knockdown of Nrf2 had a somewhat stronger effect on the ability of sterubin to reduce IL6 production in response to IFNγ and LTA than in response to LPS. Thus, these results suggest that anti-inflammatory pathways in addition to those mediated by Nrf2 are likely to contribute to the inhibition of NO and IL6 production by sterubin in microglial cells exposed to either LTA or IFNγ.

## 4. Discussion

The key observation from this study is that not only is sterubin highly effective at protecting cells from oxytosis/ferroptosis and other related and unrelated forms of cell death, it also has strong anti-inflammatory activity against multiple pro-inflammatory stimuli reducing both NO and cytokine production. In contrast, most of the other structurally closely related flavonoids that we tested were either much less effective or ineffective in both assays at the concentrations that we used. OHGK stood out as an outlier because, while it was highly neuroprotective, it had little or no anti-inflammatory activity which we tied to its inability to induce functional Nrf2.

There is increasing evidence that flavonoids could be useful for the treatment of AD e.g., [21,22]. Importantly, a recent epidemiological study found that people with the highest consumption of flavonoids had a significantly reduced risk of developing AD as well as AD-related dementias (ADRD) [23]. Representatives of all of the six classes of flavonoids have been shown to ameliorate at least some aspects of AD in animal models [21,22]. These include improvements in cognitive function, reductions in synapse loss, decreases in Aβ pathology, and reductions in markers of both inflammation and oxidative stress. The precise signaling pathways by which flavonoids mediate these beneficial effects are still being explored but induction of Nrf2, as we show here for sterubin, and activation of the ERK pathway, an indicator of increased neurotrophic factor activity, have been seen in multiple studies [21,22].

Importantly, our results clearly illustrate how very small changes in the structure of flavonoids can have highly significant effects on their bioactivity. Furthermore, they indicate that these activities are not necessarily consistent across multiple assays. Based on the structure–activity relationship from the results of the HT22 cell experiments, it is apparent that the catechol group on the B ring plays a key role in the protective effects of the compounds against oxytosis/ferroptosis as well as IAA toxicity since all of the compounds that failed to protect in these assays lack a catechol group either because one of the two hydroxyl groups is absent (sakuranetin) or because it is blocked with a methyl group (homoeriodictyol, hesperetin, chrysoeriol). The A ring 7-methoxy group also appears to enhance the protective effects since the two compounds that have this group (sterubin and OHGK) are significantly more effective than the two, otherwise identical, compounds that lack it (eriodictyol and luteolin, respectively), and this could be explained in part by their improved lipophilicity and cell permeability which result in the achievement of higher potencies (Table 3). However, conjugation of the C ring does not seem to have a significant effect on protective activity against oxytosis/ferroptosis.

Interestingly, most of the flavonoids were much more effective against Aβ toxicity in the MC65 cells despite the observation that there are significant overlaps between Aβ toxicity and oxytosis/ferroptosis [10,24]. Sterubin, eriodictyol, and luteolin which all contain a catechol group with an *ortho* dihydroxyl on the B ring had EC_50_s ~10-fold lower for protection in the MC65 cells as compared with the HT22 cells treated with glutamate, erastin, RSL3, or IAA. Surprisingly, OHGK was much less effective in this assay although the reasons for this are unclear at this time. Chrysoeriol, homoeriodictyol, and hesperetin also provided low micromolar levels of protection in the MC65 cell assay. In contrast to sakuranetin which showed very poor protection in this assay, chrysoeriol, homoeriodictyol, and hesperetin all contain an *ortho* hydroxy-methoxy pair on the B ring suggesting that this structural characteristic could contribute to their protective effects against Aβ toxicity.

While the structural requirements for protection against oxytosis/ferroptosis are quite clear, those for protection against H_2_O_2_ toxicity appear more complex. H_2_O_2_ is known for its cytotoxicity due to the generation of highly reactive hydroxyl radicals (a type of reactive oxygen species, ROS) via the Fenton reaction in cells, which is catalyzed by intracellular Fe^2+^ ions [25]. Flavonoids, however, are thermodynamically favorable for scavenging ROS, including H_2_O_2_-derived hydroxyl radicals, by hydrogen and electron donations from their phenolic hydroxyl groups leading to the formation of conjugated and stable semiquinone and/or quinone structures [26,27]. Although the catechol group clearly appears to be important for the activity of sterubin and the related flavonoids against H_2_O_2_, other structural characteristics also seem to play a role. For example, a 7-methoxy group on the A ring also seems to contribute to protection against H_2_O_2_ since both sterubin and OHGK were more effective against H_2_O_2_ than the equivalent compounds lacking the 7-methoxy group (eriodictyol and luteolin, respectively). Curiously, while conjugation of the C ring slightly reduced protection by OHGK as compared to sterubin, it seemed to increase protection by luteolin as compared to eriodictyol.

Surprisingly, the results of the studies on anti-inflammatory activity are also not as clear cut. While the catechol group seems to play an important role in the anti-inflammatory activity of the flavonoids, the role of the A ring 7-methoxy group is much less clear (sterubin vs eriodictyol, OHGK vs luteolin). Furthermore, while conjugation of the C-ring does not seem to have a significant effect on anti-inflammatory activity (luteolin vs eriodictyol), the combination of this conjugation with the A ring 7-methoxy (OHGK vs sterubin) abrogates anti-inflammatory activity which appears to be tied to the inability to induce active Nrf2.

Since we previously showed that the protective effects of sterubin against inducers of oxytosis/ferroptosis in the HT22 cells were partially dependent on Nrf2 [7], we looked at the ability of the related flavonoids to induce nuclear Nrf2 and downstream signaling in these cells to determine if there was a correlation between Nrf2 induction and protection from cell death. However, contrary to what we expected, we found that only sterubin and eriodictyol were able to activate Nrf2 in the HT22 cells even though OHGK was as effective as sterubin at protecting the cells from the inducers of oxytosis/ferroptosis while luteolin showed a similar efficacy to eriodictyol. Thus, these results indicate that while the conjugation of the C ring does not affect protective activity, it does affect the ability to induce active Nrf2. Furthermore, these data implicate other pathways in the protective effects of OHGK and luteolin. For example, luteolin has been shown to inhibit both the JNK and p38 MAPK pathways which have been implicated in cell death [28]. However, further studies are needed to understand the signaling pathways underlying the potent protective effects of OHGK.

We showed in an earlier study [7] that the anti-inflammatory effects of sterubin were highly dependent on its ability to activate Nrf2 in the BV2 cells. Consistent with its lack of effect on Nrf2 in the HT22 cells, OHGK also did not induce nuclear Nrf2 in the BV2 cells strongly suggesting that its lack of anti-inflammatory activity was due to its inability to induce this transcription factor. Curiously, luteolin showed a level of anti-inflammatory activity similar to that seen with eriodictyol although, like OHGK, it also did not induce nuclear Nrf2. This suggests that, unlike OHGK, luteolin is able to activate other pathways that can contribute to its anti-inflammatory activity.

While studies in other types of cells have shown that some of the flavonoids that we found were unable to induce nuclear, active Nrf2 including homoeriodictyol [29], sakuranetin [30], chrysoeriol [31], and hesperetin [32] were able to activate Nrf2 in other cell types, our observations serve to reinforce the idea of cell type dependent Nrf2 activation by flavonoids. The mechanisms underlying these effects are unknown. In contrast, the published data on luteolin and Nrf2 are mixed with some studies showing induction of Nrf2 [33] and others demonstrating inhibition of Nrf2 activation by luteolin [34,35]. In addition, sakuranetin [36], chrysoeriol [37], and hesperetin [38] have all been reported to have anti-inflammatory activity although this was at concentrations (25–100 µM) well above those tested in our study.

Interestingly, we also found that the distinct neuroprotective and anti-inflammatory activities of sterubin and the seven structurally related flavonoids are independent of their overall physiochemical properties. As shown in Table 3, all eight flavonoids tested generally meet the desirable multiparameter criteria to be viable CNS drug candidates in terms of their molecular weight (MW), calculated partition coefficient (CLogP), topological polar surface area (TPSA), number of hydrogen-bond donors (HBDs), and number of hydrogen-bond acceptors (HBAs). The small variations in their physiochemical parameters are attributed to the minor structural changes in the flavonoids resulting from either the addition of an *O*-methyl group or removal of a hydroxyl group on the A and B rings, or the presence of a conjugated double bond on the C ring. In general, such structural modifications can subtly increase the lipophilicity and rigidity of flavonoids and thus make them more cell membrane permeable for better intracellular target engagement. However, among the three *O*-methylated flavanones (sterubin, homoeriodictyol and hesperetin) that possess essentially the same physicochemical properties, sterubin stood out as by far the most potent compound in our screens (Table 1 and Table 2). Therefore, our structure-activity relationship analyses further support stereo- and regiospecific structural requirements rather than physicochemical attributes as the main contributors to the distinct and potent bioactivities of sterubin.

In a series of recent studies [16,17,39], Drs. Taguchi and Kunisada and their colleagues have looked at the effects of sterubin and related flavonoids on hair graying. Consistent with our results on the neuroprotective and anti-inflammatory activities of sterubin and related flavonoids, they found that sterubin or extracts of *Eriodictyon* sp. that were highly enriched in sterubin were able to prevent radiation-induced damage to normal human epithelial keratinocytes and to promote melanin production in melanocyte stem cells [16,39]. Further studies [17] that looked at the regeneration of pigmented hair after wounding in mice showed that only sterubin and related flavonoids (luteolin, OHGK and eriodictyol) that contain a catechol group were able to promote regeneration of both pigmented and non-pigmented hairs. In contrast, homoeriodictyol, hesperetin, and diosmetin which all contain a catechol group that is blocked by methylation had no effect on the regeneration of either pigmented or non-pigmented hairs.

**Figure 5:**
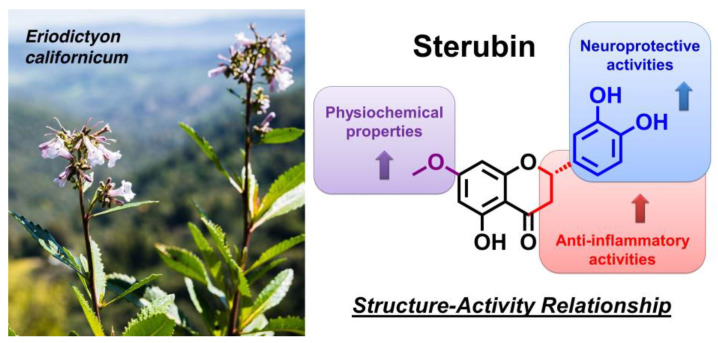
A summary of structural requirements for the neuroprotective and anti-inflammatory activities of sterubin identified in the plant *Eriodictyon californicum*.

In summary, using our robust battery of novel cell-based phenotypic screening assays, our systematic studies comparing sterubin to seven structurally closely related flavonoids highlight how the specific combination of modifications to the A, B, and C rings in sterubin leads to a unique compound that is highly protective against multiple insults related to aging and AD and also has strong anti-inflammatory activity. From a medicinal chemistry perspective, these results also further emphasize how very small structural changes in flavonoids can have dramatic effects on their activity against distinct insults. Together, the structure-activity relationship data highlighted in Figure 5 provide additional support and mechanistic guidance for the further development of sterubin as a potential neuroprotective drug candidate.

## Figures and Tables

**Table 1:** Protective effects of sterubin and related flavonoids in HT22 and MC65 cells.

Compound	Protection against Glutamate EC_50_ (µM)	Protection against Erastin EC_50_ (µM)	Protection against RSL3 EC_50_ (µM)	Protection against IAA EC_50_ (µM)	Protection against H_2_O_2_ EC_50_ (µM)	Inhibition of Aβ Toxicity EC_50_ (µM)
Sterubin	0.8	0.5	0.7	0.9	4.7	0.07
Eriodictyol	2.9	2	10	5	>10	0.31
Homoeriodictyol	>10	>10	>10	>10	>10	3.1
Hesperetin	>10	>10	>10	>10	>10	4.4
Sakuranetin	>10	>10	>10	>10	>10	>10
Hydroxygenkwanin	0.3	0.2	0.3	0.5	7.3	2.4
Chrysoeriol	>10	>10	>10	>10	>10	8.1
Luteolin	2	2	4	2	8.9	0.35

For all of the insults tested, the EC_50_s for protection by each of the compounds are indicated in the columns. All of the assays except for the protection against Aβ toxicity were done using HT22 cells. The Aβ protection assay used MC65 cells. >10 means that there was some inhibition of cell death at 10 µM but less than 50% so that the EC_50_ was above 10 µM.

**Table 2:** Anti-inflammatory effects of sterubin and related flavonoids in BV2 cells.

Compound	LPS NO EC_50_ (µM)	LPS IL6 EC_50_ (µM)	LPS IL1β EC_50_ (µM)	LTA NO EC_50_ (µM)	LTA IL6 EC_50_ (µM)	LTA IL1β EC_50_ (µM)	IFNγ NO EC_50_ (µM)	IFNγ IL6 EC_50_ (µM)
Sterubin	1.3	0.92	1.0	0.9	0.8	0.3	4.2	4.8
Eriodictyol	4.1	9.4	>10	5.5	6.9	6.3	>10	No
Homoeriodictyol	No	No	No	>10	9.7	>10	No	No
Hesperetin	No	No	No	>10	>10	No	No	No
Sakuranetin	>10	>10	No	>10	>10	No	>10	>10
Hydroxygenkwanin	>10	>10	No	>10	7.8	8	>10	4.2
Chrysoeriol	No	No	No	>10	>10	No	>10	9.9
Luteolin	5.7	8.6	7.2	6.5	5.6	>10	>10	10

For all of the pro-inflammatory stimuli tested (LPS, LTA, IFNγ) the EC_50_s for the inhibition of the production of the pro-inflammatory products (NO, IL6, IL1β) by each of the compounds are indicated in the columns. No indicates that there was no inhibition of pro-inflammatory product production at concentrations up to the highest concentration tested (10 µM). >10 means that there was some inhibition of pro-inflammatory product production at 10 µM but less than 50% so that the EC_50_ was above 10 µM.

**Table 3:** Physiochemical properties of flavonoids.

Compound	MW	ClogP	TPSA (Å^2^)	HBDs (n.OH,NH)	HBAs (n.O,N)
Desired CNS druglikeness *^a^*	≤360	≤3	40–90	≤3	≤7
Sterubin	302	2.37	96	3	6
Eriodictyol	288	1.85	107	4	6
Homoeriodictyol	302	2.29	96	3	6
Hesperetin	302	2.29	96	3	6
Sakuranetin	286	2.97	76	2	5
Hydroxygenkwanin	300	2.90	96	3	6
Chrysoeriol	300	2.75	96	3	6
Luteolin	286	2.31	107	4	6

*^a^*Desired CNS druglikeness should present molecular weight (MW) ≤360, calculated partition coefficient (CLogP) ≤3, topological polar surface area (TPSA) 40–90, number of hydrogen-bond donors (HBDs) ≤3, and number of hydrogen-bond acceptors (HBAs) ≤7 to improve their penetration of the blood-brain barrier (BBB).

## Data Availability

All the data is contained within the article.
